# Improvement of Peptide-Based Tumor Immunotherapy Using pH-Sensitive Fusogenic Polymer-Modified Liposomes

**DOI:** 10.3390/molecules21101284

**Published:** 2016-09-26

**Authors:** Yuta Yoshizaki, Eiji Yuba, Toshihiro Komatsu, Keiko Udaka, Atsushi Harada, Kenji Kono

**Affiliations:** 1 Department of Applied Chemistry, Graduate School of Engineering, Osaka Prefecture University, 1-1 Gakuen-cho, Naka-ku, Sakai, Osaka 599-8531, Japan; ss108086@edu.osakafu-u.ac.jp (Y.Y.); yuba@chem.osakafu-u.ac.jp (E.Y.); harada@chem.osakafu-u.ac.jp (A.H.); 2 Department of Immunology, School of Medicine, Kochi University, Kohasu, Okou-cho, Nankoku, Kochi 783-8505, Japan; tkomatsu@kochi-u.ac.jp (T.K.) udaka@kochi-u.ac.jp (K.U.)

**Keywords:** peptide vaccine, pH-sensitive liposome, immunotherapy, dendritic cell, pH-sensitive polymer

## Abstract

To establish peptide vaccine-based cancer immunotherapy, we investigated the improvement of antigenic peptides by encapsulation with pH-sensitive fusogenic polymer-modified liposomes for induction of antigen-specific immunity. The liposomes were prepared by modification of egg yolk phosphatidylcholine and l-dioleoyl phosphatidylethanolamine with 3-methyl-glutarylated hyperbranched poly(glycidol) (MGlu-HPG) and were loaded with antigenic peptides derived from ovalbumin (OVA) OVA-I (SIINFEKL), and OVA-II (PSISQAVHAAHAEINEAP_β_A), which bind, respectively, to major histocompatibility complex (MHC) class I and class II molecules on dendritic cell (DCs). The peptide-loaded liposomes were taken up efficiently by DCs. The peptides were delivered into their cytosol. Administration of OVA-I-loaded MGlu-HPG-modified liposomes to mice bearing OVA-expressing E.G7-OVA tumors induced the activation of OVA-specific CTLs much more efficiently than the administration of free OVA-I peptide did. Mice strongly rejected E.G7-OVA cells after immunization with OVA-I peptide-loaded MGlu-HPG liposomes, although mice treated with free OVA-I peptide only slightly rejected the cells. Furthermore, efficient suppression of tumor volume was observed when tumor-bearing mice were immunized with OVA-I-peptide-loaded liposomes. Immunization with OVA-II-loaded MGlu-HPG-modified liposomes exhibited much lower tumor-suppressive effects. Results indicate that MGlu-HPG liposomes might be useful for improvement of CTL-inducing peptides for efficient cancer immunotherapy.

## 1. Introduction

Recent developments in tumor immunology have attracted much attention to cancer immunotherapy, a treatment to activate the patient’s own immunity against tumors or remove immunosuppression in tumor microenvironments. The induction of cancer-specific cytotoxic T lymphocytes (CTLs) is crucially important to achieve efficient therapeutic effects because CTLs can kill tumor cells directly [1,2,3]. Actually, adoptive transfer of tumor-infiltrating T-lymphocytes causes marked therapeutic effects in cancer patients [4]. Earlier studies show that CTLs recognize the complex of cancer antigen and major histocompatibility complex (MHC) class I molecules expressing on cancer cells [5,6]. Furthermore, a tumor-specific mutant antigen designated as neoantigen plays an important role for the recognition of cancer cells by CTLs in actual cancer [7,8,9]. Therefore, identification of neoantigen-derived cancer antigenic peptides and the induction of these peptide antigen-specific CTLs are important to establish personalized cancer treatments using peptide-based cancer vaccines.

Since the discovery of melanoma antigen MAGE, various tumor antigenic peptides have been reported, such as WT1, glypican-3, MART-1, and TRP-1 [1,10,11,12]. Moreover, numerous clinical trials using these antigenic peptides have been attempted [3,13,14,15]. However, their therapeutic efficacy in clinical trials is insufficient even though tumor antigenic peptides have specificity to a tumor and the ability to induce antigen-specific immune responses in vitro [2]. Therefore, although tumor antigenic peptide-based immunotherapy is eagerly sought, it is still under development.

**Figure 1:**
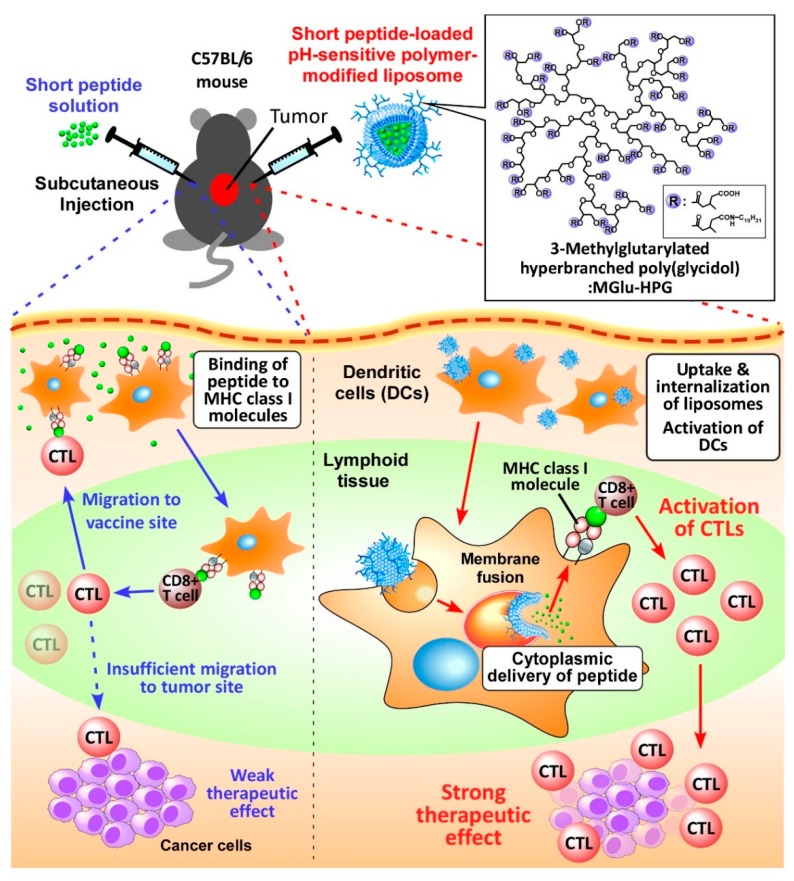
Schematic illustration of antigen delivery routes for free peptide and peptide-loaded pH-sensitive polymer-modified liposomes.

Generally, peptide-based cancer vaccine is conducted using antigenic peptides emulsified in incomplete Freund’s adjuvant (IFA) [1]. After subcutaneous administration of peptide/IFA, peptides are released gradually from IFA emulsion and are bound to MHC molecules by displacement of endogenous peptides on antigen-presenting cells (APCs) such as dendritic cells (DCs), macrophages, and B cells. These peptides can also be taken up by APCs. Peptides are bound to MHC class I molecules via cross presentation or MHC class II molecules in endosome/lysosome. APCs present antigenic peptides to CD8^+^ T cells and CD4^+^ T cells, which differentiate, respectively, to antigen-specific CTLs and helper T cells [5,6]. Subsequently, CTLs migrate to tumor sites and induce tumor-killing effects. Helper T cells support the CTL’s tumor killing effect and B cell activation. The antigen processing pathway of peptides in antigen-presenting cells and immune-inducing process are known to be affected by the peptide length. Especially, immunization with antigenic short peptides emulsified in IFA induced trapping of CTLs at original vaccine sites [16,17]. Short antigenic peptides are presented continuously to CD8^+^ T cells through MHC molecules, not only on DCs but also on B cells in peptide/IFA injected sites because peptide/IFA emulsion remains at the injected site. Continuously released short peptide molecules bind to MHC molecules on APCs. Such a peptide presentation by APCs induces the accumulation of CTLs at the peptide-injected site with high peptide concentration, which suppresses migration of CTLs into the tumor site (Figure 1). CTLs accumulated at the peptide vaccine-injected site lose their activity and undergo apoptosis, consequently producing poor antitumor effects [17].

This fact suggests the importance of effective antigen presentation by DCs for the induction of CTLs which can migrate into tumor sites and which can eliminate tumor cells effectively. Therefore, to achieve efficient immunotherapy with short tumor antigenic peptides, it is crucially important to deliver them into DCs specifically and efficiently without excessive presentation for APCs of other kinds. Then, the short peptides taken up by DCs at vaccination sites might be presented on DCs and induce antigen-specific CTLs that can migrate into tumors (Figure 1). Therefore, such DC-specific peptide delivery systems are expected to improve the efficacy of short peptide vaccine for cancer immunotherapy.

To date, carrier systems of various types have been examined for their feasibility for delivery of short antigenic peptides for the induction of antigen-specific immunity. For example, amphiphilic polyethyleneimine-based micelles were used for delivery of Trp2 peptide, which is an antigenic peptide of melanoma [18]. After subcutaneous administration, micelles accumulate preferentially to lymph nodes, probably because of their small size (around 30 nm) and induced Trp2-specific CTLs in vivo [18]. Additionally, poly(dl-lactide-*co*-glycolide) (PLGA)-based polymeric nanoparticles loaded with short antigenic peptides of MART-1, gp100 or ovalbumin (OVA) have been shown to induce stronger antigen-specific CTLs and antitumor effects than IFA/peptide emulsion [19]. However, these systems might present shortcomings in toxicity derived from cationic polymers, peptide-loading capability and controllability of peptide delivery in the body or inside of APCs. Indeed, liposomes are promising systems for short peptide delivery from the perspectives of biocompatibility, high peptide-loading capability, and functionalization, which enhance the accuracy of peptide delivery to DCs after administration.

We previously developed liposomes modified with pH-sensitive fusogenic polymer MGlu-HPG (Figure 1) and demonstrated that the MGlu-HPG liposomes have excellent capability for induction of antigen-specific immunity using OVA as antigenic protein [20,21,22,23]. In fact, MGlu-HPG-modified liposomes delivered antigenic protein OVA into cytosol of DCs via membrane fusion responding to weakly acidic pH of the endosomes/lysosomes and induced MHC class I-restricted antigen presentation [20,21,22,23]. Administration of these liposomes to OVA-expressing tumor-bearing mice induced OVA-specific cellular immunity and induced efficient suppression of tumors of mice [23]. In addition, these liposomes were taken up by DCs efficiently through the recognition by scavenger receptors of DCs because liposome surfaces are covered with many carboxylates derived from MGlu-HPG [20,21,22,23]. Based on these results, in this study, we attempted to apply the pH-sensitive polymer-modified liposomes to short peptide vaccine delivery to DCs because we expected that the liposomes might solve the problem of the short peptide vaccine, trapping of CTLs at the vaccination sites, by delivering the short peptides into cytosol of DCs and inducing antigen-specific CTLs effectively (Figure 1).

## 2. Results and Discussion

### 2.1. Preparation of Peptide-Loaded Liposomes and Selection of Adjuvant

**Figure 2:**
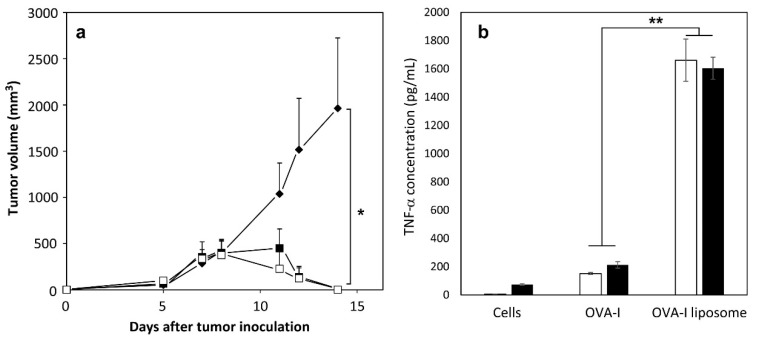
(**a**) Antitumor effect induced by immunization with MGlu-HPG-modified liposomes with or without Wc on tumor-bearing mice. The E.G7-OVA cells (1 × 10^6^ cells) were inoculated subcutaneously into the left back of C57BL/6 mice and MGlu-HPG-modified liposomes with Wc (closed squares) or without Wc (open squares) containing 50 μg of OVA were administered subcutaneously into the backs of the mice on Day 5. Mice immunized with PBS (closed diamonds) are shown as controls. Each treated group included four mice; * *p* < 0.01; (**b**) TNF-α production from DC2.4 cells treated with 10 μg of OVA-I or OVA-I-loaded MGlu-HPG-modified liposomes with (closed bars) or without Wc (open bars) for 4 h in the absence of serum; ** *p* < 0.05.

For the induction of efficient immune responses, both antigen delivery into DCs and the activation of DCs should be achieved simultaneously. In an earlier study, monophosphoryl lipid A (MPLA), which stimulates Toll-like receptor 4 (TLR4) [24], has been introduced to MGlu-HPG-modified liposomes to activate DCs [23]. MGlu-HPG-modified liposomes without adjuvant induced antitumor immunity. Their therapeutic effect was enhanced further by inclusion of MPLA [23,25]. In this study, *B. pertussis* whole cell vaccine (Wc), which is known to induce Th1 immune responses [26,27], was compared with MPLA as another adjuvant. The effect of Wc and MPLA inclusion to MGlu-HPG liposomes on the induction of antigen-specific antitumor immunity was examined (Figure 2a). PBS- or OVA-loaded MGlu-HPG-modified liposomes and 1 × 10^7^ cells of Wc were administered subcutaneously to E.G7-OVA tumor-bearing mice. For mice administered PBS, the E.G7-OVA tumor volume increased rapidly after Day 5 (Figure 2a). However, mice administered the MGlu-HPG-modified liposomes containing MPLA or a combination with liposomes and Wc exhibited decreased E.G7-OVA tumor volume after Day 8 (Figure 2a), which indicates that OVA-specific immunity was induced by administration of the OVA-loaded MGlu-HPG-modified liposomes. That result is consistent with the results of our earlier study [23]. The combination of Wc did not affect the antitumor effects induced by MGlu-HPG-modified liposomes containing MPLA. In both cases, E.G7-OVA tumor burden disappeared at Day 14, which indicates that antitumor immunity induced by MGlu-HPG-modified liposomes was sufficient to regress the established tumor.

**Figure 3:**
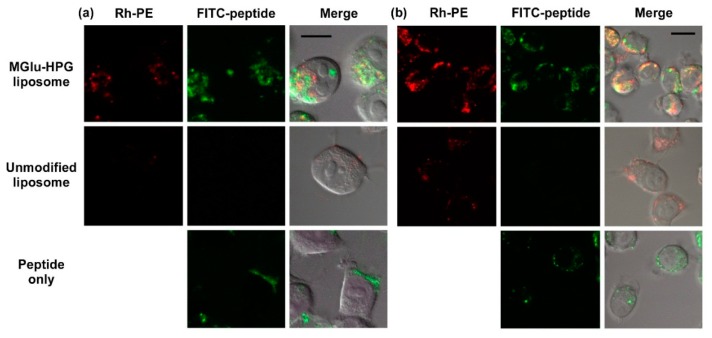
Confocal laser scanning microscopic (CLSM) images of DC2.4 cells treated with FITC-OVA-I peptides (**a**); FITC-OVA-II peptides (**b**); and peptide-loaded EYPC/DOPE/Rh-PE liposomes modified with or without MGlu-HPG. Cells were treated with peptide or liposomes with peptide concentration of 25 μg/mL for 4 h in the absence of serum. Scale bars represent 10 μm.

Next, the activation of DC2.4 cells by treatment with free peptide or peptide-loaded pH-sensitive polymer-modified liposomes was examined. OVA-I peptide (SIINFEKL)-loaded MGlu-HPG liposomes were prepared. Their diameters were about 100 nm according to dynamic light scattering (DLS) analysis (Figure S1a). A spherical structure for liposomes was also found using transmission electron microscopic (TEM) analysis (Figure S1b). Loading amounts of peptides in the liposomes were about 30 μg/μmol lipid (Figure S1c). The loading efficiency of peptide to liposome was 42.5% ± 1.5% for unmodified liposome and 44.5% ± 4.5% for MGlu-HPG liposome. MGlu-HPG liposomes retained encapsulated peptide at pH 7.4, but released at acidic pH (Figure S1d). DC2.4 cells were treated with free OVA-I or OVA-I-loaded MGlu-HPG liposomes containing MPLA with or without Wc for 4 h. Furthermore, the production of TNF-α by DC2.4 cells was measured. Figure 2b shows that production of TNF-α was enhanced by OVA-I-loaded MGlu-HPG liposomes, irrespective of the presence of Wc compared with free OVA-I solution. This result indicates that MPLA-introduced liposome treatment strongly activated DC2.4 cells, which might derive from the activation via MPLA and efficient intracellular delivery of OVA-I to DC2.4 cells (Figure 3a). Therefore, we used MPLA-introduced MGlu-HPG-modified liposomes in the following experiments considering their ease of inclusion as a lipid membrane component.

### 2.2. Intracellular Distribution of Peptide-Loaded Liposomes

Next, intracellular peptide delivery performance of MGlu-HPG-modified liposomes was investigated. DC2.4 cells were treated with FITC-labeled peptide and peptide-loaded liposomes labeled with lissamine rhodamine B-sulfonyl phosphatidylethanolamine (Rh-PE). Then they were observed using confocal laser scanning microscopy (CLSM) (Figure 3). For free OVA-I peptides, most green fluorescence was observed at the cell periphery, which indicates that FITC-labeled peptides were bound mainly to the cellular membrane (Figure 3a). For cells treated with unmodified liposomes, punctate red fluorescence was observed within cells, although green fluorescence was not observed under experimental conditions. This result suggests that unmodified liposomes are not taken up efficiently by DCs. Therefore, these liposomes were ineffective for delivery of OVA-peptides to the cytosol. For MGlu-HPG liposomes, strong red or green fluorescence was observed within the cells. In addition, some green fluorescence was found at different locations from red fluorescence. This result suggests that MGlu-HPG-modified liposomes were taken up efficiently by DCs through recognition by scavenger receptors on DCs, as reported previously [20,21,22,23]. Their contents (OVA-peptides) were delivered into the cytosol of DCs through membrane fusion responding to acidic pH of endosomes [20,21,22,23]. Efficient delivery of short peptides into the interior of DCs by MGlu-HPG-modified liposomes is important to avoid antigen presentation by other APCs at the injected site after administration to mice. In addition, some OVA-I peptides were delivered to cytosol, which induces MHC class I-mediated presentation and the induction of cellular immune response. OVA-II peptides (PSISQAVHAAHAEINEAP_β_A) were also delivered efficiently to the interior of DCs by MGlu-HPG-modified liposomes as well as OVA-I peptides (Figure 3b). To evaluate the endosomal escape efficiency, the colocalization ratio of green fluorescence with red fluorescence was calculated from CLSM images (Figure S2). As Figure S2 shows, about 50% of green fluorescence was merged with red fluorescence, indicating that about 50% of FITC-peptides exist in endosome/lysosome, and 50% exist in cytosol. For OVA-II peptides, release in endosomes or lysosomes is necessary for MHC class II presentation [5,6]. Therefore, efficient antigen presentation via MHC class II pathway might be expected by MGlu-HPG liposomes from peptides released in endosome/lysosome.

### 2.3. Induction of Cellular Immune Responses in Vivo

**Figure 4:**
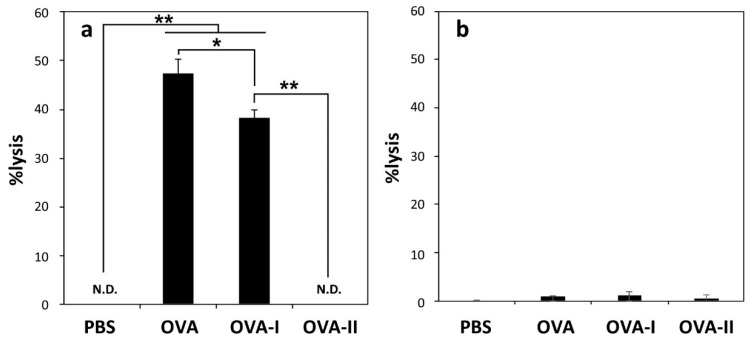
CTL response in spleen seven days after subcutaneous immunization with: (**a**) 50 μg of OVA-loaded, OVA-I-loaded, and OVA-II-loaded MGlu-HPG-modified liposomes; and (**b**) 50 μg of free OVA, OVA-I, or OVA-II. Cytotoxicity against E.G7-OVA cells was measured using an LDH assay at an effector cells/target cell (E/T) ratio of (**a**) 5 or (**b**) 1. Each bar represents means ± SD (*n* = 3). * *p* < 0.05, ** *p* < 0.01. No significant difference was found between any groups in (**b**).

The induction of CTLs is crucially important for establishing tumor regression. Therefore, the induction of CTL in spleen was measured for mice immunized with liposomes. Figure 4a and Figure S3a depict the percent lysis for E.G7-OVA cells or EL4 cells induced by the stimulated splenocytes at E/T ratio of 5. Splenocytes obtained from mice treated with OVA-I-loaded MGlu-HPG liposomes induced CTL response as well as OVA-loaded MGlu-HPG liposomes. In contrast, splenocytes obtained from mice treated with OVA-II-loaded MGlu-HPG liposomes induced no detectable CTL responses under experimental conditions. This result is reasonable because OVA-II peptide induces MHC class II-mediated presentation, which induces helper T cells, not CTLs. These splenocytes exhibited no cytotoxicity against EL4 cells. Therefore, CTLs induced by OVA-I-loaded MGlu-HPG liposomes were specific to OVA expression on the cells. Moreover, the administration of free OVA or free OVA-peptides showed quite weak CTL response in the spleen (Figure 4b and Figure S3b). These results suggest that OVA-I-loaded MGlu-HPG liposomes can induce antigen-specific CTLs efficiently because of their efficient cytoplasmic delivery of OVA peptide to DCs (Figure 3a). Therefore, OVA-I-loaded liposomes are expected to induce tumor-specific therapeutic effects against E.G7-OVA tumor-bearing mice.

### 2.4. Induction of Antitumor Responses

**Figure 5:**
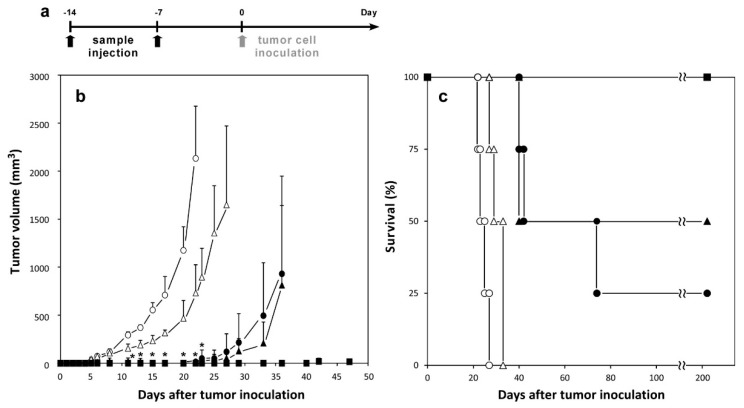
Induction of OVA-specific immunity by immunization with MGlu-HPG-modified liposomes containing OVA proteins or OVA peptides on mice. C57BL/6 mice were subcutaneously immunized with 50 μg of OVA-I solution (open circles), OVA-I-loaded MGlu-HPG-modified liposomes (closed circles), OVA-II solution (open triangles), OVA-II-loaded MGlu-HPG-modified liposomes (closed triangles), and OVA-loaded MGlu-HPG-modified liposomes (closed squares) 14 and seven days before tumor cell inoculation. Seven days after second immunization, E.G7-OVA cells (1 × 10^6^ cells) were inoculated subcutaneously into the left back of each mouse. The tumor volume was monitored. A timeline of experiments (**a**); change in tumor volume (**b**); and survival of mice (**c**) are shown. Each point represents means ± SD (*n* = 4). Mice were killed when tumor volumes became greater than 2500 mm^3^. * *p* < 0.05 compared with free peptide-treated groups. Results of Log-rank test are shown in Table S1.

The induction of antitumor immunity was investigated using MGlu-HPG-modified liposomes containing OVA peptides (Figure 5). OVA peptide-loaded liposomes were administered subcutaneously into mice and then, E.G7-OVA cells were inoculated to the mice. OVA protein-loaded MGlu-HPG liposomes were also administered as a positive control to confirm the tumor rejection by OVA-specific CTLs [20,21,22,23]. The tumor size and mice survival were monitored. As presented in Figure 5a, mice immunized with free OVA-I or OVA-II peptide solution exhibited a rapid increase in tumor volume after five days from the inoculation of tumor cells. However, mice immunized with these peptides-loaded MGlu-HPG liposomes showed a marked delay in the day on which the rapid tumor volume increase took place, indicating that the encapsulation of OVA-peptides by MGlu-HPG liposomes improved antitumor immune responses. For OVA-I-loaded MGlu-HPG liposomes, OVA-specific CTLs induced by these liposomes might kill the tumor cells efficiently (Figure 4a). Compared to the OVA-I-loaded MGlu-HPG liposomes, OVA-II-loaded MGlu-HPG liposomes also showed tumor growth suppression, although these liposomes did not induce OVA-specific CTLs under the experimental conditions (Figure 4a). Although details of the mechanism remain unclear at present, presumably, efficient delivery of OVA-II peptide to DCs by MGlu-HPG liposomes might induce MHC class II-mediated antigen presentation and the production of Th1 cytokine such as IFN-γ from OVA-specific Th1 cells. Th1 cytokine might activate NK cells or other innate immune cells to attack the tumor cells, resulting in tumor cell rejection. Survival rates of tumor-inoculated mice with immunization are presented in Figure 5b. Encapsulation of OVA-peptides by MGlu-HPG liposomes caused a significant improvement of the survival rates compared with administration of OVA-peptide solution (*p* = 0.00673 for OVA-I, *p* = 0.00912 for OVA-II, Table S1). Actually, 25% or 50% mice survived, respectively, for longer than 200 days by the immunization with the liposome-encapsulated OVA-I or OVA-II peptide. Therefore, the use of MGlu-HPG liposomes enables the improvement of antigenic short peptides for the induction of antitumor immunity. Although the reason why a subset of mice treated with peptide-loaded MGlu-HPG liposomes survived remains unclear at present, individual differences of mice might affect the difference of mouse survival.

### 2.5. Therapeutic Effects of Peptide-Loaded Liposomes on Tumor-Bearing Mice

**Figure 6:**
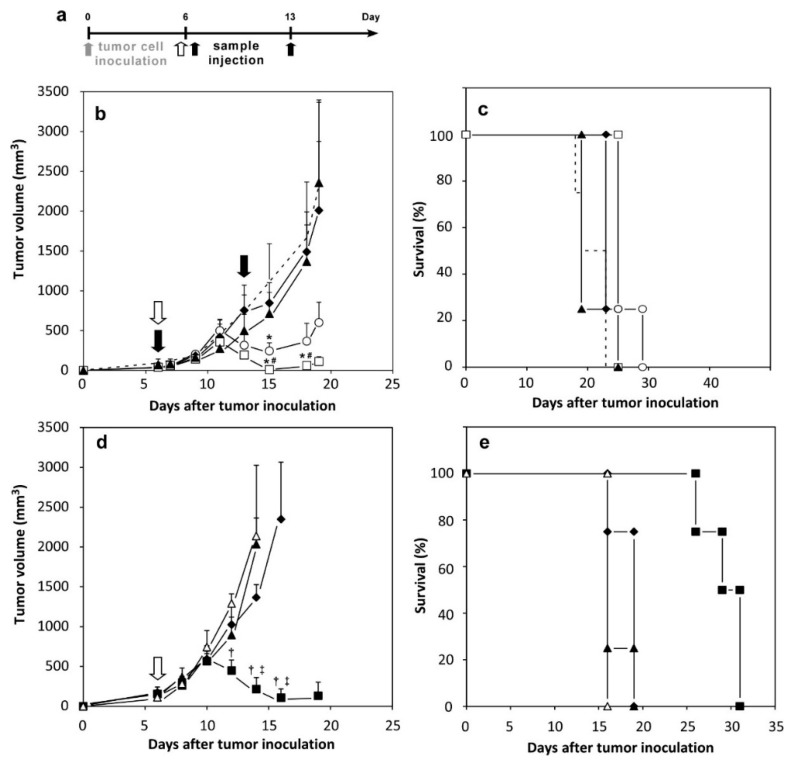
Antitumor effects induced by immunization with MGlu-HPG-modified liposomes containing OVA proteins or OVA peptides on tumor-bearing mice. (**a**) Timeline of experiments; (**b**,**c**) The E.G7-OVA cells (5 × 10^5^ cells) were inoculated subcutaneously into the left back of C57BL/6 mice and MGlu-HPG-modified liposomes containing 50 μg of OVA proteins (squares), MGlu-HPG-modified liposomes containing 50 μg of OVA-I peptides (circles), unmodified liposomes containing 50 μg of OVA-I peptides (diamonds) and OVA-I solution (triangles) were administered subcutaneously into the right back of the mice once on Day 6 (open symbols) or twice on Days 6 and 13 (closed symbols). Data for mice immunized with PBS (dotted line) are presented as control data. * *p* < 0.05 compared with PBS-treated groups. # *p* < 0.05 for unmodified liposomes-treated groups. Results of a Log-rank test are shown in Table S2; (**d**,**e**) The E.G7-OVA cells (5 × 10^5^ cells) were inoculated subcutaneously into the left back of C57BL/6 mice and MGlu-HPG-modified liposomes containing 50 μg of OVA proteins (closed squares), MGlu-HPG-modified liposomes containing 50 μg of OVA-II peptides (closed triangles) and OVA-II solution (open triangles) were administered subcutaneously into the right back of the mice on Day 6, Mice immunized with PBS (closed diamonds) are shown as controls. All treated groups included four mice. Changes in tumor volumes (**b**,**d**); and mice survival (**c**,**e**) are shown. Mice were killed when tumor volumes became greater than 2500 mm^3^. † *p* < 0.05 compared with OVA-II solution-treated groups. ‡ *p* < 0.05 compared with OVA-II-loaded MGlu-HPG-modified liposome-treated groups. Results of Log-rank tests are shown in Table S3.

Finally, we investigated the improvement of antigenic short peptides by encapsulation with MGlu-HPG liposomes for the treatment of tumor-bearing mice (Figure 6). E.G7-OVA cells were inoculated subcutaneously to mice. After six days, free or liposome-encapsulated OVA-I or OVA was injected subcutaneously into the mice. Tumor sizes were monitored from the day of inoculation. Figure 6b shows that tumor volumes of mice treated with PBS increased rapidly. However, the mice treated with OVA-loaded MGlu-HPG-modified liposomes, which were used as a positive control, showed a decrease in tumor volume after Day 12, indicative of tumor suppression induced by OVA-specific CTL [23]. Administration of free OVA-I or OVA-I-loaded unmodified liposomes showed no tumor suppression, indicating that neither free OVA-I nor OVA-I-loaded unmodified liposomes are effective for induction of antitumor immunity. In contrast, OVA-I-loaded MGlu-HPG-modified liposomes exhibited significant suppression of tumor growth after Day 12, as was the case of OVA-loaded MGlu-HPG-modified liposomes based on *p*-value. Compared to the OVA-loaded liposomes, the OVA-I-loaded liposomes exhibited a lower degree of tumor suppression, probably because OVA protein has higher antigenicity than short peptide OVA-I. Effects of boost immunization were also examined. These liposomes were administered to mice twice (Days 6 and 13) (Figure S4). Then, their therapeutic efficacy was compared to that of their single administration. As presented in Figure 6b and Figure S4b, and in Table S2, the survival period increased significantly (*p* = 0.0285) by additional administration of the OVA-I-loaded MGlu-HPG liposomes.

Immunization with OVA-II-loaded MGlu-HPG liposomes before tumor cell inoculation showed tumor growth suppression (Figure 5). Therefore, we also examined therapeutic effects of OVA-II peptide-loaded MGlu-HPG-modified liposomes (Figure 6d,e, Table S3). However, the OVA-II-loaded liposomes exhibited tumor suppressive effects only slightly. Indeed, OVA-II peptide was designed to be presented on MHC class II, which engenders the induction of OVA-specific helper T cells, not CTL.

Therefore, it is likely that the MGlu-HPG liposomes achieved efficient association to DCs through interaction with scavenger receptors and efficient delivery of OVA-I peptide into DC cytosol using their pH-sensitive membrane fusion ability [20,21,22,23]. Subsequent presentation of OVA-I peptide on MHC class I might cause efficient induction of target-specific CTL. As a result, significant tumor suppression was induced. Indeed, OVA-I peptide encapsulated with MGlu-HPG liposomes exhibited much stronger ability for induction of tumor-specific immunity and therapeutic effects than the free form of the same peptide. The immunity-inducing effect by OVA-I peptide encapsulated with MGlu-HPG liposomes remained lower than that of OVA protein-loaded MGlu-HPG liposomes, probably because OVA protein has many epitopes and can induce various CTLs and Th cells [22,23]. In the case of actual cancer treatment, limited amounts of tumor whole proteins can be recovered from patients. In contrast, tumor peptides can be synthesized to an unlimited degree, which is the most effective point of using of tumor peptides instead of whole tumor proteins. Future efforts will be undertaken to improve the immunity-inducing ability of MGlu-HPG liposomes by introduction of adjuvant functions such as cationic lipid, CpG-ODN, cytokine gene. In addition, higher antigenic long peptides have been developed for the induction of both CTL and Th cells [28]. The use of such long peptides is also expected to enhance the therapeutic effects of peptide-based vaccines.

## 3. Materials and Methods

### 3.1. Materials

Egg yolk phosphatidylcholine (EYPC) and l-dioleoyl phosphatidylethanolamine (DOPE) were kindly donated by NOF Corp. (Tokyo, Japan). Rh-PE was purchased from Avanti Polar Lipids Inc. (Birmingham, AL, USA). OVA and MPLA were purchased from Sigma (St. Louis, MO, USA). 3-Methyl-glutarylated hyperbranched poly(glycidol) with polymerization degree of 60 (MGlu-HPG, Figure 1) was used as a pH-sensitive polymer. It was prepared as explained in an earlier report [21]. OVA-I peptide (SIINFEKL) [29] and derivation of OVA-II peptide (PSISQAVHAAHAEINEAP_β_A), a modified I-A^d^-binding OVA epitope peptide described elsewhere [30], were used as model short antigenic peptides that bind respectively to MHC class I and II molecules on DCs. These peptides were synthesized using Fmoc chemistry as previously reported [26]. The peptides were purified by reverse phase HPLC to a purity of >95%. Their molecular weights were confirmed using MALDI-TOF mass spectrometry (Voyager DE–RP; Applied Biosystems, Carlsbad, CA, USA). The peptide concentration of was determined using a Micro BCA assay (Pierce, Carlsbad, CA, USA). FITC-labeled peptides were purchased from KareBay TM Biochem, Inc. (Monmouth Junction, NJ, USA). *Bordetella pertussis* whole cell vaccine (Wc), which is a suspension of inactivated bacteria in PBS, was produced from *B. pertussis* Tohama phase I bacteria and provided by Osaka University (Bikenkai, Japan).

### 3.2. Liposome Preparation

For liposome preparation, 1.0 mL of peptides/PBS solution (pH 7.4, 1 mg/mL) was added to a dry, thin membrane of EYPC (5.0 mg), DOPE (4.7 mg), and MPLA (50 μg). The mixture was vortexed at 4 °C. The liposome suspension was hydrated further by freezing and thawing. It was extruded through a polycarbonate membrane with 100 nm pore size. The liposome suspension was centrifuged at 55,000 rpm for 2 h at 4 °C twice to remove free peptides from the peptide-loaded liposomes. MGlu-HPG-modified liposomes were also prepared according to the procedure described above using dry membrane of a lipid mixture with MGlu-HPG (lipids/polymer = 7/3, *w*/*w*). OVA whole protein-loaded liposomes were also prepared according to the procedure described above using OVA/PBS solution (pH 7.4, 4 mg/mL). The concentrations of lipids, peptides and proteins were determined using Wako phospholipids C (Wako Pure Chemical Inds. Ltd., Osaka, Japan), Micro BCA assay and Coomassie (Bradford) protein assay kit (Pierce Biotechnology Inc., Carlsbad, CA, USA), respectively.

### 3.3. Cell Culture

DC2.4 cells, which were an immature murine DC line, were provided by K. L. Rock (Harvard Medical School, Worecester, MA, USA) and were grown in RPMI-1640 (Nacalai Tesque Inc., Kyoto, Japan) supplemented with 10% FBS (MP Biomedicals, Santa Ana, CA, USA), 2 mM l-glutamine (Wako, Osaka, Japan), 100 mM MEM nonessential amino acid (Nacalai Tesque Inc.), 50 μM 2-mercaptoethanol (2-ME; Gibco, Carlsbad, CA, USA), 100 U/mL penicillin, and 100 μg/mL streptomycin at 37 °C [31]. EL4, a C57BL/6 mice-derived T lymphoma, was obtained from Tohoku University (Sendai, Japan). E.G7-OVA, a chicken egg OVA gene-transfected clone of EL4 that presents OVA with MHC class I molecules, was obtained from the American Type Culture Collection (Manassas, VA, USA) [29].

### 3.4. Animals

Female C57BL/6 mice (H-2^b^, 7 weeks old) were purchased from Oriental Yeast Co. Ltd. (Tokyo, Japan). The experiments were conducted in accordance with guidelines for animal experimentation at Osaka Prefecture University.

### 3.5. Intracellular Behavior of Liposomes

The FITC-labeled OVA peptide-loaded liposomes containing Rh-PE were prepared as described above except that a mixture of polymer and lipid containing Rh-PE (0.1 mol %) was dispersed in PBS containing FITC-OVA peptides (1 mg/mL). DC2.4 cells (3 × 10^5^ cells) cultured for 2 days in 35-mm glass-bottom dishes were washed with Hank’s balanced salt solution (HBSS). Then they were incubated in serum-free RPMI medium (1 mL). The FITC-OVA peptide-loaded liposomes (50 μg/mL peptide concentration, 1 mL) were added gently to the cells and were incubated for 4 h at 37 °C. After incubation, the cells were washed three times with HBSS. CLSM analysis of these cells was performed using LSM 5 EXCITER (Carl Zeiss Co., Oberkochen, Germany).

### 3.6. Cytokine Production from Cells Treated with Liposomes

The DC2.4 cells (4 × 10^4^ cells) cultured overnight in 96-well plates were washed with HBSS. Then they were incubated in serum-free RPMI medium (100 μL). OVA-I solution or OVA-I-loaded MGlu-HPG liposomes with/without Wc (4 × 10^4^ cells) (100 μg/mL peptide concentration, 100 μL) were added gently to the cells, followed by incubation for 4 h at 37 °C. After incubation, supernatants of cultured cells were collected for measurements of TNF-α using an enzyme-linked immunosorbent assay kit (ELISA Development Kit, PeproTech EC Ltd., Rock Hill, NJ, USA) according to the manufacturer’s instructions.

### 3.7. CTL Assay

Mice were administered PBS, 50 μg of OVA-loaded MGlu-HPG liposomes, OVA-I-loaded MGlu-HPG liposomes, OVA-II-loaded MGlu-HPG liposomes, free OVA, OVA-I, and OVA-II solution subcutaneously. Seven days later, splenocytes were recovered from each mouse and were suspended in RPMI1640 supplemented with 10% FBS, 100 U/mL penicillin, 100 μg/mL streptomycin, 50 μM 2-ME, and 20 U/mL recombinant murine IL-2 (Peprotech Inc.). Splenocytes were then stimulated with mitomycin C-treated E.G7-OVA cells at a ratio of 10:1 for 5 days. The stimulated splenocytes were used as effector cells for the cytotoxicity assay. The CTL activity after 4 h-incubation was evaluated at a ratio of effector cells to target cells (E.G7-OVA or EL4 cell), which was defined as the E/T ratio, of 5 using a lactate dehydrogenase (LDH) cytotoxicity detection assay (Takara Bio Inc., Shiga, Japan).

### 3.8. Induction of Antitumor Immunity by Liposomes

OVA-loaded or OVA-peptide-loaded liposomes and free OVA or OVA peptide solution (50 μg of protein or peptide) were administered subcutaneously into the right back of C57BL/6 mice 14 and 7 days before tumor cell inoculation under anesthesia with isoflurane. Seven days after the second immunization (Day 0), E.G7-OVA cells (1 × 10^6^ cells) were inoculated subcutaneously into the left back of anesthetized mice. The tumor size was monitored using the following formula: (major axis × minor axis^2^) × 0.5 and survival of the mice was also measured. Mice were killed when tumor volumes become greater than 2500 mm^3^. Each treated group included four mice.

### 3.9. Therapeutic Effects Induced by Liposomes on Tumor-Bearing Mice

E.G7-OVA cells (5 × 10^5^ cells) were inoculated subcutaneously into the left back of C57BL/6 mice under anesthesia with isoflurane. On Days 6 and/or 13, 50 μg of OVA-loaded or OVA-peptide-loaded liposomes and free OVA or OVA peptide solution were injected subcutaneously into the right back of the mice under anesthesia. Tumor sizes were monitored from the day of inoculation. Mice immunized with PBS were used as controls to confirm the development of cancer following the first inoculation with E.G7-OVA cells. Mice were killed when tumor volumes become greater than 2500 mm^3^. Each treated group included four mice.

### 3.10. Statistical Analysis

Tukey–Kramer method (Figure 2, Figure 4, Figure 5 and Figure 6, and Figures S1–S4) was used for statistical evaluation of the results. Log-rank tests were applied for the statistical analysis of survival data (Figure 5c, Figure 6c,e and Figure S4b).

## 4. Conclusions

This study investigated the improvement of antigenic short peptide OVA-I through their encapsulation with MGlu-HPG-modified liposomes, which have both a high affinity to DC and pH-sensitive membrane fusion ability. Indeed, we observed that encapsulation of the peptide in the liposomes strongly improved the immune-induction ability of the peptide. Immunization with the liposome-encapsulated peptide achieved growth suppression of established tumors and complete rejection of tumors in a subset of mice. Results demonstrate that the use of appropriate carriers such as MGlu-HPG liposomes, which deliver short peptide vaccines into DC cytosol efficiently, can greatly potentiate their efficacy. Therefore, MGlu-HPG liposomes offer great potential to improve the efficacy of peptide vaccine for antigen-specific CTL, which is crucially important for the establishment of efficient cancer immunotherapy.

## Supplementary Materials

Supplementary materials can be accessed at: http://www.mdpi.com/1420-3049/21/10/1284/s1.
