# Supplementary material for: Improvement of Peptide-Based Tumor Immunotherapy Using pH-Sensitive Fusogenic Polymer-Modified Liposomes
Source: Molecules. 2016 Sep 26;21(10):1284. doi: 10.3390/molecules21101284 (PMC6274290; doi:10.3390/molecules21101284)
Supplement: Supplementary file 1 [file molecules-21-01284-s001.pdf]

# Supporting Information: Improvement of Peptide-Based Tumor Immunotherapy Using pH-Sensitive Fusogenic Polymer-Modified Liposomes

**Yuta Yoshizaki, Eiji Yuba, Toshihiro Komatsu, Keiko Udaka, Atsushi Harada and Kenji Kono**

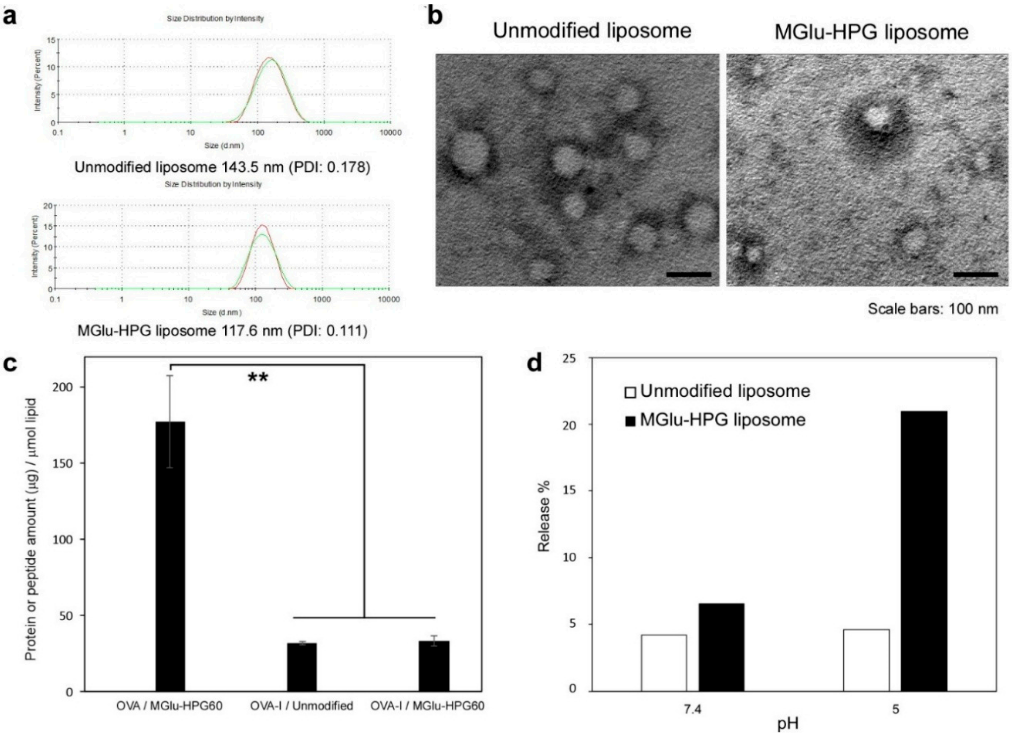


**Figure S1.** (**a**) Diameters of peptide-loaded liposomes with or without MGlu-HPG determined by Dynamic light scattering (DLS); (**b**) TEM image for OVA-I-loaded liposomes with or without MGlu-HPG; (**c**) Peptide or protein amounts per lipid in various liposomes. ** $p < 0.01$; (**d**) Peptide release from liposomes. Liposomes were incubated at pH 7.4 or 5.0 for 30 min and then liposome suspension were centrifuged and peptide amounts in supernatant were determined by Micro BCA assay.

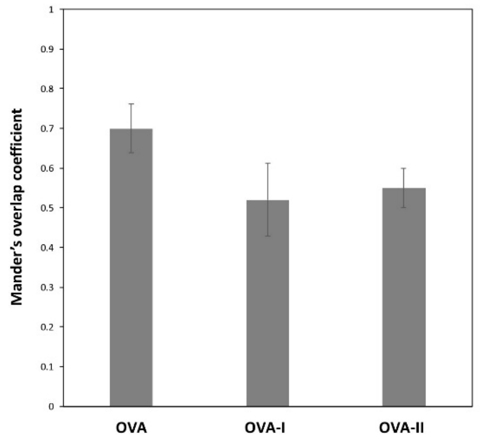


**Figure S2.** Colocalization for FITC fluorescence derived from FITC-OVA or FITC-peptide with rhodamine fluorescence. Mander's Overlap Coefficient of FITC fluorescence with rhodamine fluorescence was calculated from CLSM images. No significant difference was found between any groups.

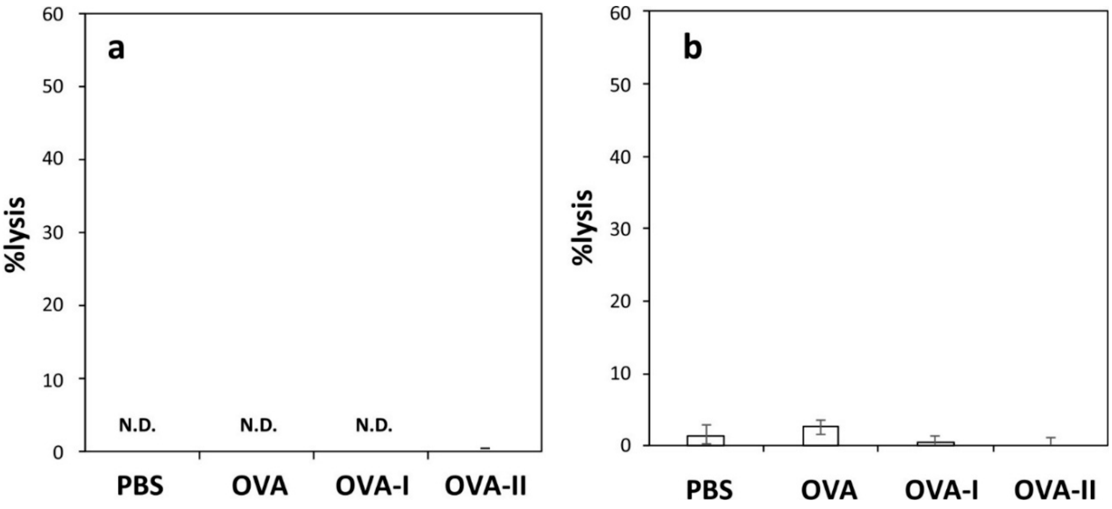


**Figure S3.** CTL response in spleen 7 days after subcutaneous immunization with (**a**) 50 µg of OVA-, OVA-I-, OVA-II-loaded MGlu-HPG-modified liposomes and (**b**) 50 µg of free OVA, OVA-I or OVA-II. Cytotoxicity against EL4 cells was measured by a LDH assay at effecter cells/target cell (E/T) ratio of (**a**) 5 or (**b**) 1. Each bar represents means ± SD ($n$ = 3). No significant difference was found between any groups according to Tukey-Kramer method.

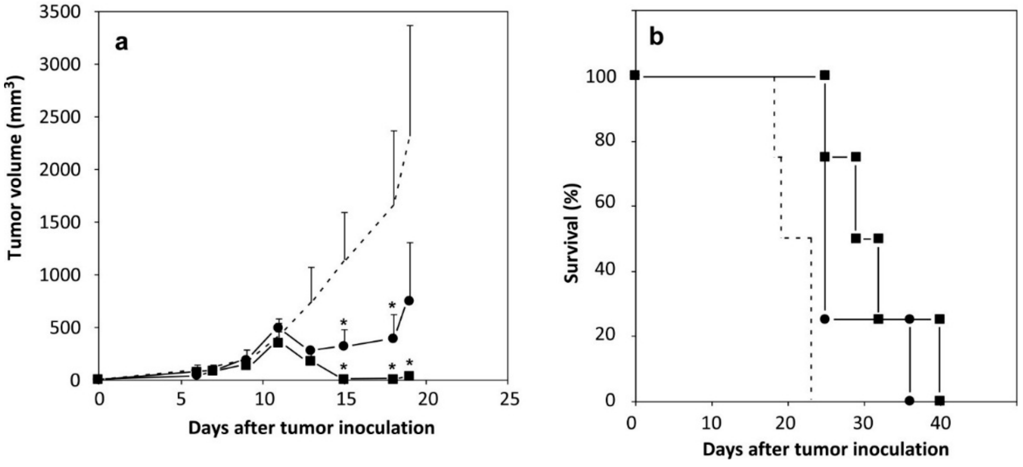


**Figure S4.** Antitumor effect induced by immunization with MGlu-HPG-modified liposomes containing OVA proteins or OVA-I peptides on tumor-bearing mice. The E.G7-OVA cells ($5 \times 10^5$ cells) were subcutaneously inoculated into the left backs of C57BL/6 mice and MGlu-HPG-modified liposomes containing 50 µg of OVA proteins (squares), MGlu-HPG-modified liposomes containing 50 µg of OVA-I peptides (circles), were subcutaneously administered into the right backs of the mice twice on days 6 and 13. Mice immunized with PBS (dotted line) were shown as controls. Change in tumor volume (**a**) and survival of mice (**b**) were shown. Mice were sacrificed when tumor volumes became over 2500 $mm^3$. * $p < 0.05$ compared with PBS-treated group. Results of Log-rank test was shown in Table S2.

**Table S1.** Survival analysis by log-rank test for Figure 5c.

| Comparison | *p* Value |
| --- | --- |
| OVA-I solution vs. **OVA-I/MGlu-HPG liposome** | 0.00673 ** |
| OVA-I solution vs. **OVA-II solution** | 0.0169 * |
| OVA-I solution vs. **OVA-II/MGlu-HPG liposome** | 0.00673 ** |
| OVA-I solution vs. **OVA/MGlu-HPG liposome** | 0.00673 ** |
| **OVA-I/MGlu-HPG liposome** vs. OVA-II solution | 0.00912 ** |
| OVA-I/MGlu-HPG liposome vs. OVA-II/MGlu-HPG liposome | 0.693 |
| OVA-I/MGlu-HPG liposome vs. **OVA/MGlu-HPG liposome** | 0.0401 * |
| OVA-II solution vs. **OVA-II/MGlu-HPG liposome** | 0.00912 ** |
| OVA-II solution vs. **OVA/MGlu-HPG liposome** | 0.00912 ** |
| OVA-II/MGlu-HPG liposome vs. OVA/MGlu-HPG liposome | 0.127 |

* $p < 0.05$, ** $p < 0.01$.

**Table S2.** Survival analysis by log-rank test for Figures 6c and S4b.

| Comparison | *p* Value |
| --- | --- |
| PBS vs. **OVA-I/MGlu-HPG liposome (once)** | 0.00912 ** |
| PBS vs. **OVA-I/MGlu-HPG liposome (twice)** | 0.00912 ** |
| PBS vs. OVA-I/unmodified liposome (twice) | 0.0971 |
| PBS vs. **OVA/MGlu-HPG liposome (once)** | 0.00912 ** |
| PBS vs. **OVA/MGlu-HPG liposome (twice)** | 0.0285 * |
| PBS vs. OVA-I solution (twice) | 0.645 |
| OVA-I/MGlu-HPG liposome (once) vs. **OVA-I/MGlu-HPG liposome (twice)** | 0.0285 * |
| OVA-I/MGlu-HPG liposome (once) vs. OVA-I/unmodified liposome (twice) | 0.645 |
| OVA-I/MGlu-HPG liposome (once) vs. OVA/MGlu-HPG liposome (once) | 0.544 |
| OVA-I/MGlu-HPG liposome (once) vs. **OVA/MGlu-HPG liposome (twice)** | 0.0415 * |
| OVA-I/MGlu-HPG liposome (once) vs. OVA-I solution (twice) | 0.645 |
| **OVA-I/MGlu-HPG liposome (twice)** vs. OVA-I/unmodified liposome (twice) | 0.0415 * |
| OVA-I/MGlu-HPG liposome (twice) vs. OVA/MGlu-HPG liposome (once) | 0.315 |
| OVA-I/MGlu-HPG liposome (twice) vs. OVA/MGlu-HPG liposome (twice) | 0.553 |
| **OVA-I/MGlu-HPG liposome (twice)** vs. OVA-I solution (twice) | 0.0415 * |
| OVA-I/unmodified liposome (twice) vs. **OVA/MGlu-HPG liposome (once)** | 0.0404 * |
| OVA-I/unmodified liposome (twice) vs. **OVA/MGlu-HPG liposome (twice)** | 0.0446 * |
| OVA-I/unmodified liposome (twice) vs. OVA-I solution (twice) | 0.307 |
| OVA/MGlu-HPG liposome (once) vs. OVA/MGlu-HPG liposome (twice) | 0.158 |
| **OVA/MGlu-HPG liposome (once)** vs. OVA-I solution (twice) | 0.0404 * |
| **OVA/MGlu-HPG liposome (twice)** vs. OVA-I solution (twice) | 0.0292 * |

* $p < 0.05$, ** $p < 0.01$.

**Table S3.** Survival analysis by log-rank test for Figure 6e.

| Comparison | *p* Value |
| --- | --- |
| PBS vs. **OVA/MGlu-HPG liposome** | 0.01 * |
| PBS vs. OVA-II/MGlu-HPG liposome | 0.774 |
| PBS vs. **OVA-II solution** | 0.0404 * |
| **OVA/MGlu-HPG liposome** vs. OVA-II/MGlu-HPG liposome | 0.0171 * |
| **OVA/MGlu-HPG liposome** vs. OVA-II solution | 0.00815 ** |
| OVA-II/MGlu-HPG liposome vs. OVA-II | 0.317 |

* $p < 0.05$, ** $p < 0.01$.
